# Suboptimal child healthcare practices and the development of multiple infectious diseases in children aged 24–59 months

**DOI:** 10.3389/fpubh.2024.1340559

**Published:** 2024-03-05

**Authors:** Ning Sulistiyowati, Dwi Hapsari Tjandrarini, Christiana Rialine Titaley, Bertha J. Que, Puti Sari Hidayangsih, Suparmi, Sudikno Sudikno, Yuni Purwatiningsih, Lely Indrawati, Selma Siahaan, Windy Pradita Adyarani

**Affiliations:** ^1^Research Center for Public Health and Nutrition, National Research and Innovation Agency, Bogor, Indonesia; ^2^Faculty of Medicine, Pattimura University, Ambon, Indonesia; ^3^Bekasi General Hospital, Bekasi, Indonesia

**Keywords:** infectious diseases, handwashing practice, supplemental food, nutritional status, type of residence, Indonesia basic health research

## Abstract

**Background:**

Infections continue to be a major cause of death among children under the age of five worldwide. This study aimed to identify the factors associated with the development of multiple infectious diseases in children aged 24–59 months in Indonesia.

**Methods:**

Data from the 2018 Basic Health Research conducted by the Ministry of Health, Republic of Indonesia, were used. Information from 39,948 children aged 24–59 months was analyzed. The outcome variable was the development of multiple infectious diseases, that is, acute respiratory infections, pneumonia, pulmonary tuberculosis, diarrhea, and hepatitis, in the month before the survey. Factors significantly associated with multiple types of infectious diseases were examined using logistic regression.

**Results:**

The study found that 76.6% of children aged 24 to 59 months in Indonesia had at least one type of infectious disease. The likelihood of developing multiple types of infectious diseases increased in children whose parents did not practice appropriate handwashing with soap and running water [adjusted odds ratio (aOR) = 1.16, *p* < 0.001], those who received supplemental food (aOR = 1.38, *p* < 0.001), those with poor nutritional status (aOR = 1.12, *p* < 0.001), and those living in urban areas (aOR = 1.07, *p* = 0.045).

**Conclusion:**

Improving caregivers’ awareness of adequate child healthcare practices, in addition to nutrition-sensitive and specific interventions to improve children’s nutritional status, is required to prevent children from contracting multiple types of infectious diseases.

## 1 Introduction

In 2019, approximately 5.3 million children under 5 years of age died, mostly from preventable causes (1). Globally, premature birth and birth complications (such as birth asphyxia/trauma), acute respiratory infections (ARIs), diarrhea, and malaria continue to be the leading causes of preventable death among children under 5 years of age (1, 2). In Indonesia, pneumonia (14.5%) and diarrhea (9.8%) were the leading causes of death among children aged 29 days to 11 months (3). Indonesia Basic Health Research in 2018 reported that 2.1 and 6.8% of children under 5 years of age have ever been diagnosed with pneumonia and diarrhea, respectively (4).

Multiple factors are reportedly associated with the occurrence of infectious diseases among children under 5 years of age. This includes a lack of access to and availability of health services, leading to delayed treatment and prolonged disease occurrence and severity (5, 6). Poor environmental health, such as a lack of safe water and sanitation, also contributes to child morbidity (7–9). At the same time, appropriate handwashing practice has been identified as one of the most prominent and affordable hygiene practices for reducing infectious diseases, such as diarrhea and respiratory infections (10).

Moreover, malnutrition has been reported to be the underlying reason for an increased risk of bacterial gastrointestinal and respiratory infections (11). A previous study highlighted the reciprocal association between malnutrition and infection because malnourished children are more likely to become infected, and recurrent infections frequently contribute to malnutrition (12). Malnutrition makes children more vulnerable to disease and death (13). Although substantial progress has been made in reducing childhood malnutrition globally, Indonesia still faces high levels of malnutrition. The National Nutritional Survey conducted in 2021 showed that a quarter of underweight children in Indonesia were stunted (24.4%), 7.1% were wasted, and 17% were underweight (14).

Various studies have investigated the relationship between nutritional status and morbidity (12, 15, 16); however, few have explored the determinants of multiple infectious diseases among children in Indonesia. Therefore, using nationally representative data, this study aimed to examine factors associated with the development of multiple infectious diseases in children aged 24 to 59 months in Indonesia. Policymakers and program managers could use the findings to design and implement evidence-based strategies to improve children’s health status by controlling factors contributing to the development of multiple infections among children in Indonesia.

## 2 Methods

### 2.1 Data sources

Data were obtained from the 2018 Basic Health Research conducted by the Ministry of Health, Republic of Indonesia. Using the multistage sampling method, the 2018 Basic Health Research was conducted in all 34 provinces of Indonesia, representing Indonesia’s district/city level. A detailed explanation of the survey method is provided elsewhere (4).

In this analysis, only information from children aged 24–59 months was used. Children in this age range were expected to have completed their basic immunization schedule beyond their lactation period (up to 23 months of age). Information from 39,948 children aged 24–59 months was analyzed. Data were obtained from household interviews and anthropometric measurements of the children.

This analysis used two anthropometric measurements in children aged 24–59 months: body weight and height/length. During data collection, the anthropometric measurements were performed by two personnel. The first person acted as the measurer, and the second person recorded the results. Height measuring devices measured the children’s heights with a measuring capacity of 2 m and an accuracy of 0.1 cm. Body weight was measured using a digital scale with a precision of 0.1 kg. All field personnel were trained to standardize the measurement procedures in the field.

### 2.2 Outcome variable

The dependent variable in this analysis was a history of multiple infectious diseases in children aged 24–59 months. Infectious diseases examined were ARIs, pneumonia, tuberculosis (TB), diarrhea, and hepatitis. This variable was classified into two groups: *none or single type* if the child had **never** or developed **only one type** of infectious disease, and *multiple types* if the child had **two or more types** of infectious disease in the past month.

### 2.3 Independent variables

**Figure 1:**
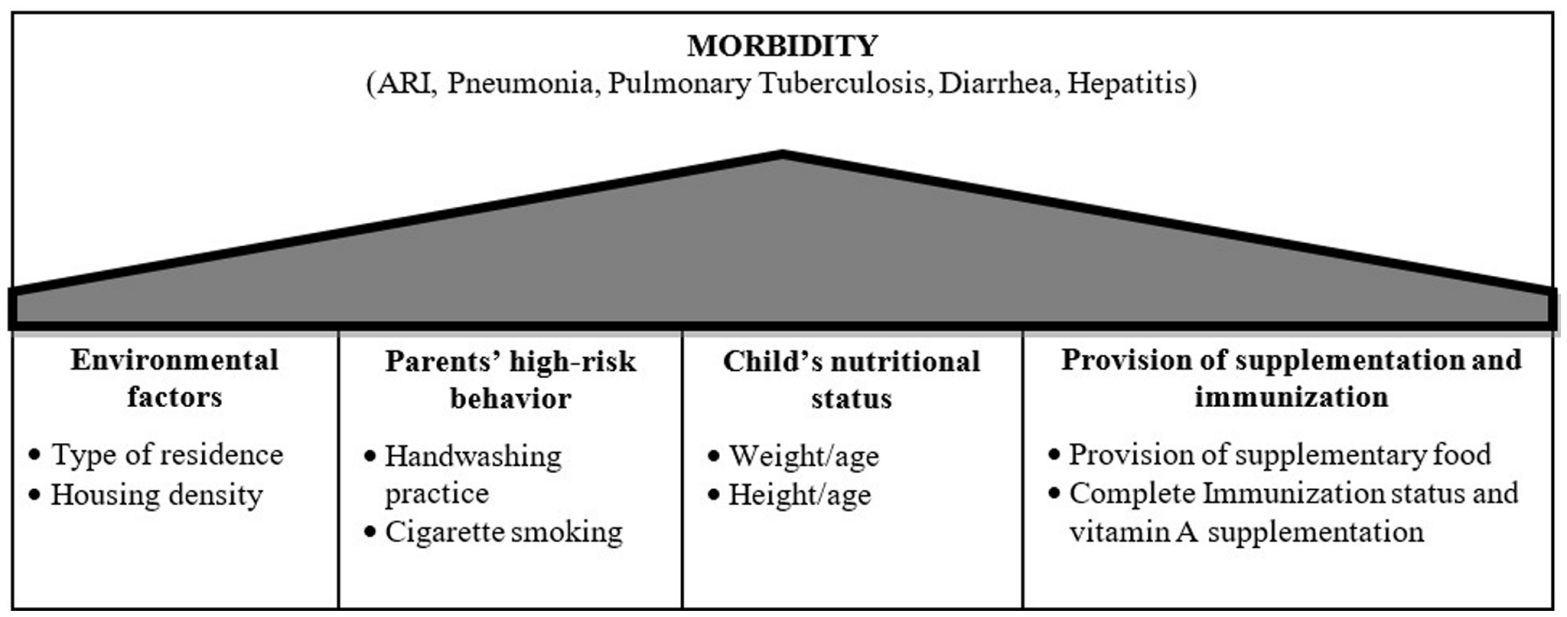
The analytical framework of factors associated with the frequent occurrence of infectious diseases in children under 5 years old in Indonesia.

Independent variables were divided into four categories: (1) environmental factors; (2) parents’ high-risk behaviors; (3) children’s nutritional status; and (4) provision of supplementation and immunization (Figure 1). Environmental factors included type of residence (urban/rural) and housing density. The housing density variable was constructed based on two criteria: a minimum housing area of 8 m^2^ per person, and the number of children under 5 years living in a house. The first category of housing density was children in the house, which met the minimum housing area criteria and had a maximum of two children in the house. The second category included children who did not meet the minimum housing area criteria or with two or more children under the age of 5 years in the house or both.

Two variables were used in a high-risk behavior group: parents’ handwashing practices and cigarette smoking behavior. Parents’ handwashing practices consisted of two categories: (1) appropriate, that is, washing hands with soap and running water; and (2) inappropriate, if otherwise. Cigarette smoking behavior also consisted of two categories: (1) both parents who had never smoked cigarettes; and (2) if otherwise.

Two variables as the children’s nutritional status indicators were used, i.e., weight/age and height/age (17). For the weight/age indicator, the children were classified into either *normal* (the *Z*-score ≥ −2.0 to *Z*-score ≤ 2.0), *overnutrition* (*Z*-score was >2.0), or *undernutrition* (*Z*-score was <−2). For the children’s height/age indicator, they were categorized as either *normal* (*Z*-score ≥ −2.0) or *short* (*Z*-score < −2) (17).

Two variables used for the provision of supplementation and immunization were: (1) provision of supplementary food (yes/no), which was one of the government programs to complement the nutritional needs of children to achieve optimal weight gain, according to their age (18); and (2) the combined childhood immunization status and vitamin A supplementation, which was based on whether the child received childhood immunization up to 11 months of age (HB0, BCG, DPT/HB/HIB 1, 2, 3, polio 1, 2, 3, 4, or IPV 1, 2, 3, and measles) (19) and vitamin A supplementation usually provided every 6 months (20). This variable consisted of the following categories: (1) received all types of childhood immunization and vitamin A supplementation; (2) received all types of childhood immunization, but never received vitamin A supplementation; (3) never received or incomplete childhood immunization and ever received vitamin A supplementation; and (4) received incomplete/never received childhood immunization and never received vitamin A supplementation.

### 2.4 Data analysis

In the first stage, data analysis was performed by examining the distribution of each variable used and then examining each potential predictor’s distribution against childhood morbidity as the outcome variable. Logistic regression analysis was used to examine the factors associated with childhood morbidity. A multivariate logistic regression analysis was performed using a stepwise method to assess the association between each potential predictor and childhood morbidity. The final model consisted of variables significantly related to the study outcome at a significance level of 0.05. The statistical software SPSS v.24, suitable for analyzing complex sample data, was used. All estimates were weighted for sampling probabilities.

### 2.5 Ethics clearance

The 2018 Basic Health Research of Indonesia was approved by the Health Research Ethics Committee of the National Institute of Research and Development, Ministry of Health, Republic of Indonesia (No.: LB.02.01/2/KE.024/2018, July 28, 2017). Informed consent was obtained from all the respondents and they signed an informed consent form before data collection.

## 3 Results

**Figure 2:**
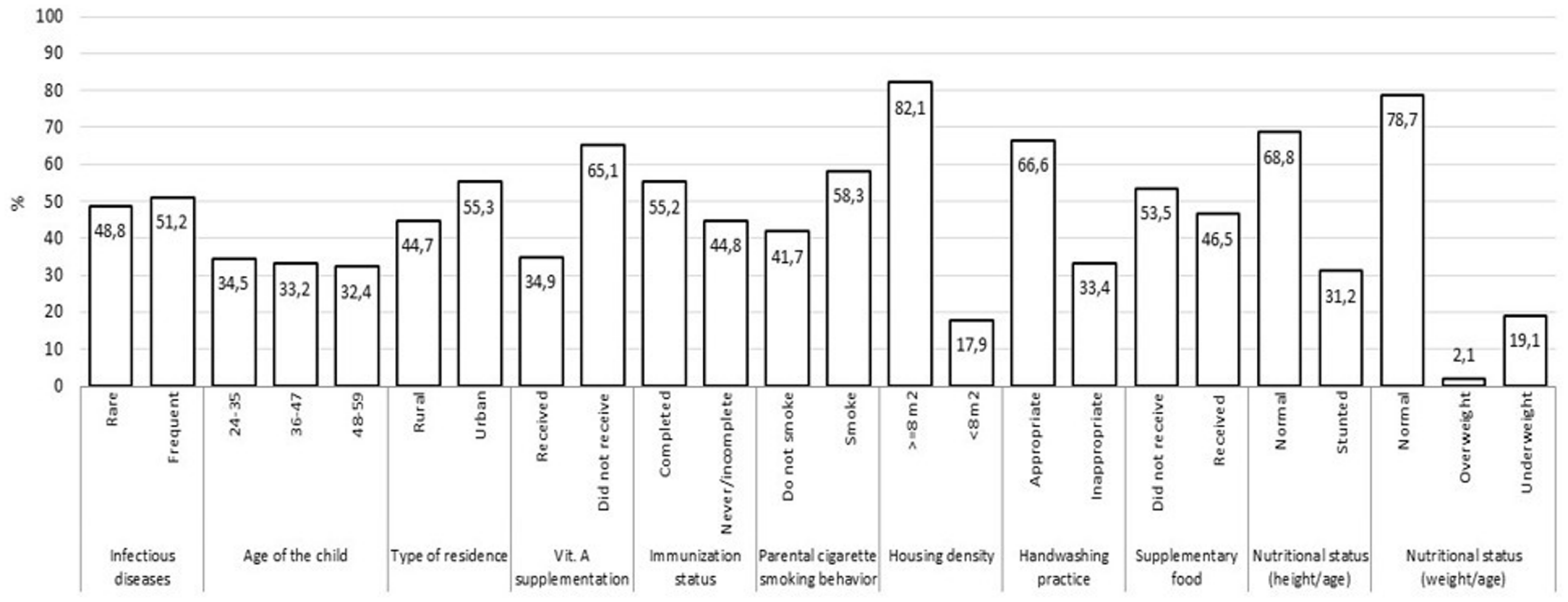
Characteristics of children aged 24–59 months, The 2018 Indonesia basic health survey.

**Figure 3:**
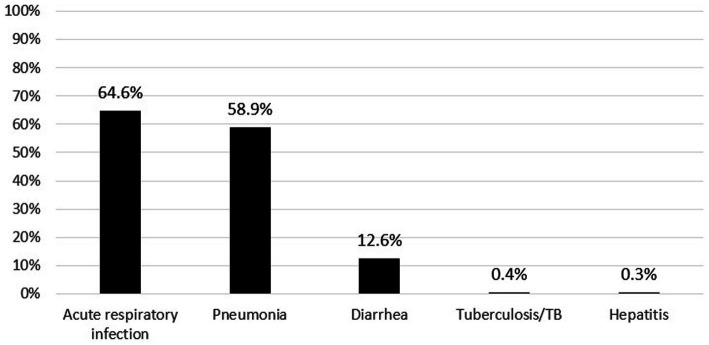
The prevalence of infectious disease in children aged 25–59 months in Indonesia, the 2018 Indonesia basic health survey.

Information from 39,948 children aged 24–59 months available from the 2018 Basic Health Research of Indonesia was used in this analysis. Figure 2 shows the characteristics of the children analyzed in this study. In the past month, 51.2% of the children had at least two types of infectious diseases. The most commonly reported infectious diseases were upper respiratory tract infections and pneumonia, whereas hepatitis and pulmonary TB were rarely reported (Figure 3). Most children lived in urban areas, >65% did not receive vitamin A supplementation, and 58.3% had parents who smoked cigarettes. Most parents reported appropriate hand-washing practices (washing their hands with soap and running water). Additionally, the prevalence of stunting and being underweight was 31.2 and 19.1%, respectively.

Table 1 shows the distribution of potential predictors based on the occurrence of infectious diseases in the past month. The percentage of children with multiple infectious diseases in the past month increased in children whose parent smoked cigarettes and those whose parents did not have proper handwashing practices. Based on bivariate analysis, the factors significantly associated with the development of multiple types of infectious diseases were handwashing practices, immunization status, vitamin A supplementation, supplemental food, and child nutrition status.

**Table 1:** Frequency distribution variables by the occurrence of infectious diseases in children under five in Indonesia, The 2018 Indonesia basic health survey.

Variable	Type of infectious disease in the past month		*n*		
	None or single	Multiple		OR	Value of *p*
	%	%			
Parental cigarette smoking behavior					0.119
Both parents ever smoked a cigarette	49.5	50.5	16,675	1.00	
Either one or both parents smoked a cigarette	48.3	51.7	23,271	1.05	
Handwashing practice					<0.001
Appropriate (with soap and running water)	49.9	50.1	26,608	1.00	
Inappropriate (another answer)	46.5	53.5	13,339	1.15	
Immunization status and vitamin A supplementation					0.028
Completed immunization and received vitamin A	49.4	50.6	6,476	1.00	
Completed immunization and never received vitamin A	48.5	51.5	15,579	1.04	
Incomplete immunization and received vitamin A	50.9	49.1	7,485	0.94	
Incomplete immunization and never received vitamin A	47.3	52.7	10,407	1.09	
Supplementary food					<0.001
Did not receive	52.6	47.4	21,366	1.00	
Received	44.5	55.5	18,581	1.39	
Nutritional status (height/age)					0.009
Normal	49.5	50.5	27,482	1.00	
Stunted	47.3	52.7	12,465	1.09	
Nutritional status (weight/age)					<0.001
Normal	49.2	50.8	31,449	1.00	
Overweight	57.4	42.6	857	0.72	
Underweight	46.2	53.8	7,641	1.13	
Housing density					0.211
8 m^2^ or above and two or fewer children under-five	49.0	51.0	32,407	1.00	
Other	47.8	52.2	7,541	1.05	
Place of residence					0.295
Rural	49.3	50.7	17,874	1.00	
Urban	48.4	51.6	22,074	1.03	
Total	48.8	51.2	39,948		

Table 2 shows four factors significantly associated with developing multiple infectious diseases in the past month after controlling for all predictors. The odds of developing multiple types of infectious diseases increased in parents who had inappropriate handwashing practices [adjusted odds ratio (aOR) = 1.16, 95% confidence interval (CI): 1.09–1.24, *p* < 0.001], children receiving supplemental food (aOR = 1.38, 95% CI: 1.30–1.47, *p* < 0.001) and in underweight children (aOR = 1.12, 95% CI: 1.04–1.20, *p* < 0.001). However, the odds were lower in children who were overweight (aOR = 0.73, 95% CI: 0.59–0.91, *p* < 0.001) than those with normal weight. Infants from urban areas also had a higher odds of developing multiple types of infectious diseases than those from rural areas (aOR = 1.07, 95% CI: 1.00–1.14, *p =* 0.045).

**Table 2:** Selected model of factors associated with frequent occurrence of infectious diseases amongst children aged 24–59 months in Indonesia, The 2018 Indonesia Basic Health Survey.

Parameter	B	aOR	95%CI		Value of *p*
(Intercept)	0,445				
Appropriate hand washing		1.00			
Inappropriate hand washing	-0,149	1.16	1.09	1.237	<0.001
Did not receive supplemental food		1.00			
Received supplemental food	-0,325	1.38	1.30	1.473	<0.001
Normal weight for age		1.00			
Overweight	-0,109	0.73	0.59	0.907	0.004
Underweight (low weight for age)	-0,419	1.12	1.04	1.200	<0.001
Rural		1.00			
Urban	-0,054	1.07	1.00	1.136	0.045

## 4 Discussion

### 4.1 Role of nutritional status in preventing infectious diseases

The importance of adequate nutrition to prevent children from infectious diseases was confirmed in this analysis. Underweight children were more likely to develop multiple infectious diseases. An underweight status usually mirrors recent and severe weight loss due to poor nutrient intake (21). This is supported by previous studies showing that undernourished children are more vulnerable to infectious diseases that adversely affect their survival (15). The relationship between undernutrition and infectious diseases is reciprocal (11, 15, 22). Undernutrition also worsens infectious diseases because it negatively affects the immune system, which is essential for protecting children from infectious agents (23). Previous studies have shown that undernourished children frequently experience respiratory infections, diarrhea, measles, and malaria, which are characterized by long and progressive disease courses (22). A Punjab-Pakistan study reported a relationship between diarrhea and underweight status in toddlers (24). Previous studies have shown that underweight children (weight/age) are more likely to experience diarrhea than normal-weight children (25, 26). A high prevalence of pneumonia among undernourished children has also been reported in Cambodia, and the duration of inpatient care due to pneumonia has increased with the increased severity of malnutrition (27). Previous literature reported that viral and bacterial skin infections were also prevalent in children (28). Insufficient nutritional status could impair the biological and structural integrity of the skin, leading to a compromised skin barrier (29). Nevertheless, due to the limited data available in the 2018 Basic Health Research, we could not incorporate skin infections into our analysis. Therefore, future studies that undertake a thorough examination of various infectious diseases, including skin infections, which are frequently observed in children are recommended.

Although several studies have reported the protective role of supplemental food in preventing child’s mortality (30) and infectious diseases (31, 32), this analysis showed that children who received supplemental food were more likely to experience multiple infectious diseases. It is highly likely that the children who received supplemental food in this study had poor nutritional status, including wasted or underweight children (33). Furthermore, several challenges have been reported in implementing this supplementary feeding program, including an unclear duration of administration (34). Poor adherence to supplementary feeding programs has emerged as an issue (35). All these conditions could contribute to an increased risk of infectious diseases among recipients of supplemental food.

These findings reflect the need for nutrition-specific and sensitive interventions that address the immediate and underlying causes of undernutrition to improve children’s nutritional status (36). Optimizing nutrition intake early in life to ensure the best start and provide long-term benefits complemented by multifaceted approaches to prevent and reduce undernutrition at the individual, household, and community levels is required (37).

### 4.2 Improving handwashing practices to prevent infectious diseases

Handwashing with soap is one of the most effective ways to prevent the transmission of infectious diseases (38). The availability of clean water is essential to support appropriate handwashing practices. The availability of clean water is associated with higher hygiene sanitation coverage and a lower risk of diarrheal diseases (39, 40). A study in India reported that washing hands before and after preparing food, defecating, and cleaning dishes significantly reduced the likelihood of diarrhea by >70% and respiratory infections by >56% (10). Mothers or caregivers who did not wash their hands at key times had an increased risk of experiencing acute diarrhea than those who practiced appropriate handwashing (41). A review reported that interventions promoting handwashing could reduce diarrhea episodes by approximately 30%, comparable to providing clean water in low-income areas (40). This indicates that improving the community’s access to handwashing facilities, soaps, and clean water is vital. This should also be complemented by interventions to promote community awareness, as mothers with sufficient knowledge would have more appropriate hand-washing practices than those with low levels of knowledge (42). Health workers should use every contact opportunity to educate mothers on the need to wash their hands with soap and running water, particularly at critical times, to reduce the risk of infection in children.

### 4.3 Importance of addressing children in urban areas

This analysis found an increased likelihood of developing infectious diseases among children living in urban areas. This aligns with previous studies that showed that children under 5 years of age living in urban areas have a higher risk of developing ARIs (43). Wahyuningsih (44) reported that the prevalence of ARI in Indonesia remained high over the years in urban areas, which was related to the physical environment of houses, including the adequacy of ventilation and sufficient sunlight in the house. Air pollution in urban areas is also associated with ARI in children (45). A previous study reported an increased risk of diarrhea in children owing to the high accessibility and availability of unsafe ready-to-eat foods with high microbial loads in urban areas (46). This emphasizes the need for health promotion program interventions to empower mothers in urban areas to make informed choices for and other caregivers about the selection and preparation of healthy food for children under 5 years old and to control various risk factors for infectious diseases related to their living environment.

### 4.4 Strengths and limitations of the study

This analysis provides national estimates of infectious diseases among children under 5 years of age in Indonesia. The large sample size used in this analysis provided adequate statistical power to examine the association between various predictors of infectious diseases. However, it is important to acknowledge several limitations of this study. Since this was a secondary analysis, our choice of variables was constrained by their availability within the dataset. Unfortunately, variables like measles or skin infections, which have been previously linked to malnutrition (29), were absent from the datasets and, consequently, had to be omitted from our analysis. The cross-sectional design did not allow the examination of potential causes and effects, but only the association between the variables examined. Another limitation was that the history of infectious diseases was based on the respondents’ recall ability and knowledge of the illnesses experienced by the children.

## 5 Conclusion

The findings show an association between inappropriate child healthcare practices (inappropriate handwashing practices and inadequate nutritional status of children) and residence type with frequent occurrence of infectious diseases in children aged 24–59 months in Indonesia. This indicates the need for multifaceted interventions to reduce infectious diseases in children under five. Effective health-promotion strategies are required for caregivers to raise awareness about the importance of appropriate childcare, including handwashing practices to prevent children from developing infectious diseases and ensuring access to clean water. Additionally, nutrition-sensitive, and nutrition-specific intervention strategies are essential to address children’s nutritional status. In urban areas, efforts to minimize the potential risk factors for infectious diseases should be a priority for health authorities and the community in general. Further research is recommended to explore different types of infections prevalent in children, including skin infections, which were not covered in our current dataset but are known to be common in this age group. This extended research could provide valuable insights for developing more targeted and effective health interventions.

## Data availability statement

The raw data supporting the conclusions of this article will be made available by the authors, without undue reservation.

## Ethics statement

The studies involving humans were approved by the Health Research Ethics Committee of the National Institute of Research and Development, Ministry of Health, Republic of Indonesia. The studies were conducted in accordance with the local legislation and institutional requirements. The participants provided their written informed consent to participate in this study.

## Author contributions

NS: Conceptualization, Writing – original draft. DT: Formal analysis, Methodology, Writing – review & editing. CT: Resources, Writing – review & editing. BQ: Resources, Writing – review & editing. PH: Formal analysis, Writing – review & editing. S: Resources, Writing – review & editing. SdS: Resources, Writing – review & editing. YP: Resources, Writing – review & editing. LI: Resources, Writing – review & editing. SeS: Writing – review & editing. WA: Writing – review & editing.
